# Low risk of thyroid eye disease (TED) following radioiodine (RAI) therapy in a multidisciplinary setting: a retrospective cohort study

**DOI:** 10.1186/s13044-025-00259-2

**Published:** 2025-08-14

**Authors:** John S. Vekinis, Kirsty Clarke, Vickie Lee, Malik Moledina, Sairah R. Khan, Mitesh Naik, Karim Meeran

**Affiliations:** 1 Western Eye Hospital, Imperial College Healthcare NHS Trust, London, UK; 2 Department of Surgery & Cancer, General Surgery, Faculty of Medicine, Imperial College London, London, UK; 3 Clinical Imaging and Nuclear Medicine, Imperial College Healthcare NHS Trust, London, UK; 4 Department of Endocrinology, Imperial College Healthcare NHS Trust, London, UK

**Keywords:** Thyroid, TED, Radioiodine, RAI, Reactivation, Risk

## Abstract

**Background:**

Radioiodine (RAI) is recommended by the National Institute of Clinical Excellence (NICE) as a first-line definitive treatment of Grave’s hyperthyroidism (GH). RAI is cited as a major risk factor in the development and exacerbation of thyroid eye disease (TED). We aim to identify the current risk of TED in a population treated post 2015.

**Methods:**

This is a retrospective cohort study including all adult patients diagnosed with GH undergoing RAI over a seven-year period. Data collected included age; gender; thyroid diagnosis and pre- and post-treatment thyroid status; length of GH; TSHR-Ab titres; smoking status; RAI dose and number of treatments; ophthalmology assessment and use of steroid prophylaxis. Investigation results, clinical scoring and management details were collected from patients who developed TED following RAI.

**Results:**

Four of 231 patients (1.7%) treated with RAI over 8 years had new onset (two patients) or progression (two patients) of TED following their RAI. One required immunosuppression and one rehabilitative TED surgery. No patient developed sight-threatening TED.

**Conclusions:**

In our study, judicious screening for high-risk patients and the use of prophylactic steroids results in lower rates of RAI associated TED relative to those reported previously in the literature.

## Background

Radioactive iodine (RAI) has proven efficacy in treating Graves hyperthyroidism (GH), representing an economical, single-administration treatment which is highly effective in controlling thyrotoxicosis [1]. It consists of an orally administered capsule containing the radioisotope iodine-131 (^131^I), which emits high energy β^−^ particles and ablates thyroid tissue via indirect and direct mechanisms resulting in DNA damage and cell death [2]. The National Institute of Clinical Excellence (NICE) and American Thyroid Association (ATA) recommend RAI as an option for first-line treatment of adults diagnosed with GH, and RAI is the most common primary therapy for uncomplicated GH in the US [1, 3–5]. However, since the 1990s, RAI has been associated with development and progression of thyroid eye disease (TED), a debilitating condition with ocular and psychosocial ramifications that limit daily functioning and vision-related quality of life [6]. It is now widely accepted that confounding variables such as smoking status, hypercholesterolaemia, and high levels of thyroid-stimulating hormone receptor antibody (TSHR-Ab) modify a patient’s risk of TED development [7]. In recent years, the extent to which RAI poses a risk of TED has been called into question, with many studies failing to reproduce the initial high complication rates of 15–20% [6, 8, 9], possibly because of careful patient selection and the use of prophylactic steroids with radioiodine.

We aim to identify the current risk of TED post-RAI therapy if administered in line with the 2021 European Group on Graves’ orbitopathy (EUGOGO) guidelines and the 2022 consensus statement published by the ATA and European Thyroid Association (ETA) [10, 11].

## Methods

Data on all patients with a diagnosis of GH undergoing RAI therapy at St Mary’s Hospital, Charing Cross Hospital, Hammersmith Hospital and West Middlesex Hospital (London, United Kingdom) over a seven-year period between May 2016 and October 2023 were analysed. GH was defined as patients with either an elevated TSHR-Ab titre or a thyroid scintigraphy study consistent with GH as per ETA and ATA Guidelines [3, 12]. RAI was administered orally as an ^131^I capsule using a fixed dose range from 400 to 600 MBq at St Mary’s Hospital or Charing Cross Hospital. Patients were advised to stop antithyroid drugs 5 to 21 days prior to RAI, with the exact interval dependent on the specific drug and its half-life. For example, carbimazole was typically stopped 5–7 days prior, whereas propylthiouracil, due to its longer duration of action, was stopped up to 21 days before treatment [3]. Patients deemed high risk for TED were reviewed by in the joint TED clinic prior to RAI and were recommended steroid prophylaxis if appropriate. The dose and duration of these steroids were included in our data collection. Post-treatment, patients were monitored with three-weekly biochemical thyroid function tests (TFTs) and oral thyroxine was commenced prior to the onset of biochemical hypothyroidism when the serum free thyroxine (FT4) concentration fell to 14nM, and before a rise in thyroid stimulating hormone (TSH) occurred. Exclusion criteria included lack of follow-up data (less than 6 months in duration) and thyroid pathology other than GH, such as an autonomously functioning thyroid nodule or toxic multinodular goitre. TED was defined as the presence of typical clinical findings diagnosed by a consultant specialist ophthalmologist and in a multidisciplinary thyroid eye clinic (MDTED) clinic examination. If the clinical diagnosis was in doubt or patients had a known previous history of TED, they underwent an MRI orbits examination which included T1, fat-saturated T2, post-contrast and diffusion-weighted sequences for radiological confirmation of TED diagnosis and to assess for signs of radiological inflammation [10, 14].

Data collected included age at RAI treatment, diagnosis of TED pre or post-RAI, duration of follow-up (time between RAI treatment and the most recent assessment by their endocrinology clinic or MDTED clinic), length of time between GH diagnosis and RAI therapy, smoking status, use of prophylactic steroids, RAI dose received. The most recent TSHR-Ab prior to RAI was recorded using an ELISA (RSRTM TRAb 3rd generation, Cardiff, UK) assay with a reference normal interval of 0.4–30 IU/L. This assay is a human monoclonal autoantibody to the TSHR, labelled with biotin, (M22-Biotin). Prior to RAI treatment, patients with suspected or confirmed TED were referred for assessment in an MDTED clinic with joint care under consultant ophthalmologists and endocrinologists. TED severity [10]radiological MRI findings, clinical activity (CAS) [15]Gorman [16]and Graves’ ophthalmopathy quality of life (GOQOL) scores [17] were recorded at each visit and treatment. The initial standard of care for all patients, with varying severity of TED, is as outlined in the contemporaneous EUGOGO, ATA and ETA (consensus) guidelines [10, 11]. Factors deemed high risk for developing or worsening TED following RAI included smoking history, previous TED diagnosis, and a high TSHR-Ab (above 5 IU/L). Steroid cover was recommended if one or more were present. We generally discouraged patients who had received prior immunosuppression for TED from RAI therapy with discussion of alternative treatment options including thyroidectomy and continuing anti-thyroid drug treatment indefinitely.

All data analysis was performed using IBM SPSS statistics 29.0.2.0. Mann–Whitney U testing was used to make comparisons between our continuous data and Fisher’s exact test for categorical data. A p-value of under 0.05 was set as significant. This retrospective audit was registered and approved by Imperial College Healthcare National Health Service (NHS) Trust, London (reference OPH_090). Data were anonymised and confidentiality was maintained throughout. This audit adhered to the tenets of the Declaration of Helsinki.

## Results

In total, 350 patients underwent RAI in the study period, though 119 were excluded as 77 had a thyroid pathology other than GH and 42 had a lack of follow-up data. We included 231 who received RAI. Four patients (1.7%) (Table 1), developed new onset or exacerbation of TED, with 3 receiving prophylactic steroid cover. There was a median period of 396.5 days (range: 109–1137, IQR: 607.25) between RAI and TED. We compared the characteristics of these four patients with the 227 who did not develop TED in Table 2.

**Table 1:** Patient characteristics of the 4 patients who developed thyroid eye disease (TED) following radioactive iodine (RAI). GH, Grave’s hyperthyroidism. TSHR-Ab, thyroid-stimulating hormone receptor antibody. CAS, clinical activity score. GOQOL VF/App, Graves’ ophthalmopathy quality of life visual function/appearance. IVMP, intravenous methylprednisolone. STIR, short tau inversion recovery

Patient	Pre-RAI TED	Smoking status at RAI	Pre-RAI TSHR-Ab (IU/L)	Ophthalmology review prior to RAI	Steroid prophylaxis	Days from prophylactic steroid to RAI	Form and dosage of steroid prophylaxis	Months from GH diagnosis to RAI	Days from RAI to TED diagnosis	MRI Findings	Number of MRIs (days since TED diagnosis)	TED management	TED scoring at TED diagnosis				Maximum TED scoring Values				EUGOGO Severity at Presentation	Maximum EUGOGO Severity
													CAS	Gorman	GOQOL VF	GOQOL App	CAS	Gorman	GOQOL VF	GOQOL App		
1	No	Current	7.8	No	No		None	32	1137	Mild unilateral proptosis	1 (1193)	Lubricant eye drops Eyelid lowering surgery Redo eyelid lowering	Not recorded				Not recorded				Moderate-to-Severe	Moderate-to-Severe
2	No	Never	25.9	Yes	Yes	3	250 mg pulsed IVMP weekly for 4 weeks	59	109	1) STIR signal in lacrimal glands and extraocular muscles. Enlargement of extraocular muscles. 2) Slight progression to muscle enlargement, continued T2 signal 3) Reduction in extraocular muscle size and no active inflammation	3 (121, 409, 1038)	IVMP Oral prednisolone Mycophenolate mofetil	4	2	100	68.75	4	2	100	31.25	Moderate-to-Severe	Moderate-to-Severe
3	Yes	Never	6.7	Yes	Yes	3	20 mg oral prednisolone daily for 1 week then reducing by 5 mg weekly (4 weeks total treatment)	18	163	Severe bilateral proptosis secondary to extraocular muscle and fat enlargement.	1 (218)	Lubricant eye drops	2	0	92.86	18.75	2	0	35.71	12.5	Mild	Moderate-to-Severe
4	Yes	Former	25.9	Yes	Yes	3	20 mg oral prednisolone daily for 2 weeks then reducing by 5 mg weekly (5 weeks total treatment)	24	630	1) No evidence of TED. 2) Rectus muscle enlargement. Increased signal in intraconal fat.	2 (665, 965)	Lubricant eye drops	2	0	100	43.75	2	0	100	31.25	Mild	Moderate-to-Severe

**Table 2:** Patient characteristics of our cohort of 231 patients split into two study groups. *Excluding 61 patients with an unknown smoking status. † Mann-Whitney U test. ‡ Fisher’s exact test. TED, thyroid eye disease. RAI, radioactive iodine. TSHR-Ab, thyroid-stimulating hormone receptor antibody

	TED	Non-TED	p-value
n	4	227	
Mean age at RAI (years)	48.5	43.0	0.473†
	(range: 34–67, SD: 16.1)	(range: 19–79, SD: 13.5)	
Median post-RAI follow-up (months)	42.5	14	**0.014**†
	(range: 22–75, IQR: 17)	(range: 0–94, IQR: 17)	
Median period from GH diagnosis to RAI (months)	28.0	36.5	0.549†
	(range: 18–59, IQR 16.2)	(range: 1-323, IQR 48.5)	
Gender			1.000‡
• Male	25% (1)	27% (62)	
• Female	75% (3)	73% (165)	
Smoking status at RAI*			0.392‡
• Current	25% (1)	16% (27)	
• Former	25% (1)	20% (34)	
• Never	50% (2)	64% (106)	
TED status at RAI			0.055‡
• Previous TED	50% (2)	10% (22)	
• No previous TED	50% (2)	90% (205)	
Median TSHR-Ab titres pre-RAI (IU/L)	16.85	3.3	**0.018**†
	(range: 6.7–25.9, IQR: 18.4)	(range: 0-34.9, IQR: 5.7)	
Median first serum free TSH following RAI (pmol/L)	0.05	0.03	0.887†
	(range: 0-16.68, IQR: 4.2)	(range: 0-128.49, IQR: 1.4)	
Thyroid status at RAI:​			0.180‡
• Hyperthyroid​	50% (2)	20% (45)	
• Euthyroid​	50% (2)	74% (168)	
• Hypothyroid	0% (0)	6% (13)	
TED Risk			0.123‡
• High	100% (4)	52% (117)	
• Low	0% (0)	48% (110)	
Median RAI dose (mCi)	695.5	594.5	**0.019**†
	(range: 622–810, IQR: 121.3)	(range: 456–861, IQR: 53.8)	
Steroid prophylaxis during RAI			**0.005‡**
• Yes	75% (3)	10% (23)	
• No	25% (1)	90% (204)	

A specialist eye examination was undertaken in 17% (39/231) of patients as they were considered high risk for development of TED, with risk factors including a previous TED diagnosis, high TSHR-Ab titres and positive smoking status; the proportion of patients with these risk factors is shown in Table 2. No patient reviewed by an ophthalmologist prior to RAI was identified to have active TED, 26 were prescribed steroid prophylaxis. The TED group had a significantly higher median TSHR-Ab titre of 16.85 IU/L (range: 6.7–25.9, IQR: 18.4) compared to 3.3 IU/L (range: 0-34.9, IQR: 5.7) in the non-TED group (*p* = 0.018). 85% of our cohort underwent RAI within one year of their GH diagnosis.

Previous diagnosis of TED was present in 20 (77%), and 4 were current smokers (15%). No patients with prior immunosuppressive TED treatment received RAI treatment. Excluding patients with an unknown steroid prophylaxis regimen, 11% (2/18) were prescribed 250 mg intravenous methylprednisolone weekly for 4 weeks starting 4 days prior to RAI and 89% (16/18) were prescribed oral prednisolone with a mean daily dose of 20.9 mg (range: 15–40, SD: 6.4), reducing over a mean length of 5.1 weeks (range: 4–8, SD 1.1). When comparing oral steroid prophylaxis regimes in the TED group and non-TED group, there was no significant difference between steroid dose (*p* = 1.000), duration of prophylaxis (*p* = 0.353) or days before RAI prophylaxis was commenced (*p* = 0.506). Table 1 describes the characteristics of patients diagnosed with TED onset or progression following RAI, for which one patient received further immunosuppression for moderate to severe disease. There were no cases of sight-threatening TED.

## Discussion

This study presents one of the largest UK-based cohorts to date examining the real-world incidence of TED following RAI treatment for Graves’ hyperthyroidism in a multidisciplinary setting Importantly, we show that even among high-risk patients, severe TED outcomes were rare and manageable, supporting the safety of RAI in well-selected individuals. Our rate of TED onset and progression post-RAI of 1.7% is significantly lower than reported over the last 30 years [18–20] and especially compared to a mean of 15% (23/150) in the landmark randomised control trial conducted by Bartalena et al. in 1998 that reported a risk of developing TED following RAI [6]. More recent studies from 2018 to 2024 have reported rates of 4% (4/101) and 5% (5/101) respectively [8, 21]. Notably, Cosentino et al. (2025), found no increased risk of Graves’ orbitopathy progression in patients treated with an ablative approach for hyperthyroidism, reinforcing the safety of RAI when combined with appropriate prophylactic strategies [22].

Bartalena et al. originally postulated that RAI exacerbates TED compared to methimazole in 1998 and observed this exacerbation could be counteracted via the prophylactic use of steroids [6]. Subsequent studies by Ponto et al. and Vannuchi et al. concurred that RAI is associated with an elevated risk of TED progression and occurrence compared to anti-thyroid drugs, recommending steroid prophylaxis in high-risk groups and to avoid post-RAI hypothyroidism [23, 24]. Current guidelines emphasise thorough ophthalmological assessment of patients considered for RAI and the administration of prophylactic steroids in moderate to high-risk groups appears to have significantly reduced the risk of TED development following RAI [10]. Steroids themselves carry inherent risks, including induction of hyperglycaemia and osteoporosis. Patients receiving steroid prophylaxis need to have a careful discussion over risks versus benefits. There are multiple different regimens recommended in the literature, however, irrespective of the regimen used, it appears that there will be a cohort who will go on to develop new TED or a TED exacerbation; which may reflect the natural history of the disease [10, 19]. Two of our cohort did develop a TED exacerbation within a 3-month period of RAI administration despite both having steroid prophylaxis though only one patient developing TED underwent RAI in the first 18 months since their GH diagnosis. We recommend a careful risk-benefit analysis when counselling patients about the best treatment options for managing GH. Those deemed high risk for TED progression would benefit from joint assessment TED multidisciplinary team (MDTED) service.

The median time from GH onset to RAI was 36 months in our cohort, reflecting the common practice in the UK of least 12–18 months of medical anti-thyroid drug therapy before definitive treatment which is also the recommendation by NICE for mild and uncomplicated GH [5]. The majority of our cohort (85%) would have passed the peak period of natural TED development of one year following GH diagnosis, as it has been previously demonstrated that around 75% of TED and GH occur within the same year of presentation. It is likely that patients who are treated with radioiodine as primary therapy for GH without a course of oral anti-thyroid drug treatment would have a higher level of apparent TED progression that may reflect the natural history of the condition. A recent study from the same part of London found a 5% rate of TED in their RAI cohort but it is not clear when these patients had RAI treatment relative to the time of GH diagnosis [21]. Two patients who developed TED after RAI were diagnosed a significantly long time following their RAI at 630 and 1137 days, this is significantly longer than the 6-month maximum time to develop TED following RAI seen by Bartalena et al. [6]. This may imply that in our patients displaying longer time periods between RAI and TED, their TED may not be a consequence of RAI. It would be difficult to differentiate between TED as a consequence of RAI and TED in a patient with history of RAI.

Despite NICE recommendations of RAI as a first-line definitive treatment for GH, in practice in the UK, most patients are started on medication and are only offered RAI in the event of persistent thyrotoxicosis or relapse following cessation of medication. In our practice, usually patients with significant previous TED requiring immunosuppression treatment opted for total thyroidectomy or lifetime carbimazole over RAI treatment following appropriate counselling and, as such, none of our cohort had previous immunosuppression for their TED. Thyroidectomy surgery in high-volume surgical centres is a safe procedure however, it does come with risks including hypoparathyroidism leading to hypocalcaemia, vocal cord paralysis and haematoma [25, 26].

The probable pathogenesis of post-RAI progression of TED is thought to be associated with the release of thyroid antigens from the radiation-damaged thyroid [27, 28]. In our study, we identified a significantly higher pre-RAI TSHR-Ab titre in our TED group when compared to our non-TED group (*p* = 0.018). TSHR-Ab has been previously demonstrated to remain increased in months to years following RAI [29].

All patients developing post-RAI TED only reached moderate-to-severe severity, maximum CAS scores were low, and only one patient required immunosuppression. Our rate of moderate-severe TED following RAI reflects a far lower rate of progression to than the 5–6% found in previous natural history studies [13, 24, 30]. Post-RAI TED may have a less severe phenotype than non-RAI-associated TED, which may be intrinsic to the RAI itself or because of close observation following RAI resulting in optimisation of modifiable risk factors.

Notably, Bartalena et al. (2023) have highlighted that the onset and progression of TED may align more closely with the natural course of Graves’ disease rather than being directly attributable to RAI therapy, particularly when RAI is administered early after disease onset. This distinction is clinically relevant, as it suggests that the perceived risk of TED related to RAI may be overstated in appropriately selected patients. Hence, earlier diagnosis and better control of risk factors support a more refined, risk-based use of RAI, potentially reducing its historic link to adverse outcomes.

There were limitations in the quality of data of this study given its retrospective nature. Smoking status at RAI was occasionally missing from patient records. Similarly, as this study was performed during the COVID-19 pandemic, patients were more likely to delay their presentation to hospital with TED symptoms or be followed up by their local centre following RAI therapy; this reduced the quantity of data available from individual patients. Importantly, shorter follow-up in the non-TED group may have led to under detection of late-onset cases. A prospective study with standardised data collection timing, steroid prophylaxis dosing, post-RAI eye examination and serialised antibody titres following RAI could provide stronger conclusions and different insights into the relationship between TED and RAI in current practice.

## Conclusion

We have found a lower rate of TED following RAI than previously reported, and no significantly increased risk in the development of TED following RAI. We have also found that post-RAI TED most frequently only reaches a moderate-severe severity, and immunosuppressive and rehabilitative treatments are rarely needed. Identification of low-risk patients, selection of patients requiring prophylactic oral steroid cover and meticulous prevention of hypothyroidism post-treatment are all essential in ensuring RAI is not only an efficacious but also safe treatment for GH patients.

## Data Availability

Data on all patients with a diagnosis of GH undergoing RAI therapy at St Mary’s Hospital, Charing Cross Hospital, Hammersmith Hospital and West Middlesex Hospital (London, United Kingdom) over a seven-year period between May 2016 and October 2023 were analysed. Data accessed from electronic healthcare records systems. The datasets generated and/or analysed during the current study are not publicly available due being healthcare data requiring approval to access from Imperial College Healthcare National Health Service (NHS) Trust. The data may be available from the corresponding author on reasonable request.

## Abbreviations

RAI: Radioiodine
NICE: National Institute of Clinical Excellence
GH: Grave’s hyperthyroidism
TED: Thyroid eye disease
ATA: American Thyroid Association
TSHR: Ab–Thyroid–stimulating hormone receptor antibody
EUGOGO: European Group on Graves’ Orbitopathy
ETA: European Thyroid Association
TFTs: Thyroid function tests
FT4: Free thyroxine 4
TSH: Thyroid stimulating hormone
MDTED: Multidisciplinary thyroid eye clinic
CAS: Clinical activity score
GOQOL: Graves’ ophthalmopathy quality of life
NHS: National Health Service
